# Analyse multicentrique de la qualité rédactionnelle des bulletins d’analyses biologiques au Niger

**DOI:** 10.11604/pamj.2022.43.59.36896

**Published:** 2022-10-07

**Authors:** Kadour Djobo, Abdourahamane Yacouba, Daouda Alhousseini, Boubacar Marou Soumana, Salao Chaibou, Mounkaila Boutchi, Saidou Mamadou

**Affiliations:** 1 Faculté des Sciences de la Santé, Université Abdou Moumouni, Niamey, Niger,; 2 Laboratoire de Biologie Médicale, Hôpital National Amirou Boubacar Diallo, Faculté des Sciences de la Santé, Université Abdou Moumouni, Niamey, Niger,; 3 Laboratoire de Biologie Médicale, Hôpital National de Niamey, Niger,; 4 Laboratoire de Biologie Médicale, Hôpital Général de Référence, Niamey, Niger,; 5 Laboratoire de Biologie Médicale, Maternité Issaka Gazobi, Niamey, Niger

**Keywords:** Non-conformité, bulletin d’analyses biologiques, Niger, Non-compliance, laboratory request forms, Niger

## Abstract

**Introduction:**

le bulletin d´analyses biologiques (BAB) constitue un moyen de communication entre le biologiste et le clinicien. Au Niger, à notre connaissance, aucune étude n´avait porté sur la qualité rédactionnelle des BAB. L´objectif de cette étude était d´évaluer la qualité rédactionnelle et le coût des BAB non conformes dans quatre principaux hôpitaux de Niamey et la représentation du laboratoire CERBA au Niger.

**Méthodes:**

il s´agissait d´une étude multicentrique, transversale et descriptive, réalisée sur une période de huit mois. Tous les BAB adressés aux laboratoires retenus durant la période d´étude ont été inclus dans cette étude.

**Résultats:**

au total, 5651 BAB provenant de 30 services cliniques différents ont été inclus dans cette étude. Les informations les plus notifiées sur les BAB étaient le nom (99,79%), prénom du patient (99,65%) et la date de prescription (97,45%). A contrario, la date, l´heure, et la nature de prélèvement étaient notifiées respectivement sur 0,02%, 0,21%, et 1,68% des BAB. Globalement, 9,45% des BAB étaient conformes aux règles de bonne prescription médicale. Si les médecins avaient tendance à prescrire mieux que les autres agents de santé à l´analyse bivariée, l´analyse multivariée avait montré que le risque des BAB non conformes n´était pas associé à la qualification du prescripteur, au service demandeur, et au coût des analyses. **Conclusion:** la qualité rédactionnelle des BAB est très faible dans les structures de santé évaluées. Ces résultats soulignent la nécessité d´un dialogue clinico-biologique et une attitude rigoureuse au niveau des laboratoires sur la gestion des non-conformités.

## Abstract (en)

**Introduction:**

laboratory request forms (LRF) is a means of communication between the biologist and the clinician. In Niger, to our knowledge, no study focused on the editorial quality of LRF. The purpose of this study was to evaluate the editorial quality and cost of non-compliant LRF in four main hospitals in Niamey and the representation of the CERBA laboratory in Niger.

**Methods:**

we conducted a multicenter, cross-sectional, descriptive study over a period of eight months. All LRFs sent to selected laboratories over the study period were included in this study.

**Results:**

a total of 5.651 LRFs from 30 different clinical departments were included in this study. The most reported information in the LRFs was: the patient’s last name (99.79%), first name (99.65%) and date of prescription (97.45%). On the other hand, the sample date, time, and nature were reported in 0.02%, 0.21%, and 1.68% of the LRFs respectively. Overall, 9.45% of the LRFs complied with the principles of good prescribing. While the bivariate analysis showed that doctors had a tendency to prescribe better than other health workers, multivariate analysis showed that the risk of non-compliance of LRFs was not associated with the prescriber’s qualification, the requesting service, and the testing cost. **Conclusion:** the editorial quality of LRFs is very low in the health structures evaluated. These results highlight the need for effective communication between the physicians and the biologists and a rigorous attitude of laboratory staff regarding the management of non-conformities.

## Introduction

Le succès de la pratique médicale moderne dépend de plus en plus de la fiabilité des résultats rendus par le laboratoire d´analyses biologiques [1]. Actuellement, plus de 70% des diagnostics médicaux sont influencés par les résultats de laboratoire [2]. Toute erreur émanant du laboratoire peut négativement impacter sur la qualité des soins fournis aux patients. Ces erreurs peuvent survenir à toutes les étapes du traitement des échantillons, notamment la phase pré-analytique, analytique et post-analytique.

Étape initiale de la phase pré-analytique, la rédaction du bulletin d´analyses biologiques amorce le début d´une analyse biologique. Le bulletin d´analyses biologiques est défini comme étant: « l´ensemble des prescriptions faites dans un but d´exploration diagnostique d´une maladie, par une autorité compétente, en l´occurrence, le médecin ou le praticien ». Les médecins en font recours pour étayer leurs diagnostics cliniques (l´anamnèse, l´examen physique). On serait tenté de dire « sans examen complémentaire, pas de traitement » [3]. Un bulletin d´analyses biologiques conforme est un bulletin répondant aux critères de rédaction des bulletins d´analyses biologiques selon la norme ISO 15 189 [4].

La prescription d´un bulletin d´analyses biologiques comporte un certain nombre de renseignements: sur le prescripteur ainsi que son service, le patient (de son identité jusqu´aux renseignements cliniques), et le prélèvement. Ceci permet de rendre l´invisible visible, de donner une meilleure interprétation des résultats, guidant le choix de la technique d´analyse et d´éviter les erreurs de manipulation, d´attribution des échantillons à d´autres patients. Cependant, en pratique courante, force est de constater l´absence de rigueur dans l´élaboration des bulletins d´analyses biologiques [5]. Cette situation aboutissait à une non-conformité, avec rejet des spécimens biologiques, à l´origine de retard de prise en charge thérapeutique et une charge de travail supplémentaire au biologiste ainsi que toutes les personnes associées à la réalisation, au traitement, et à l´interprétation des résultats de ce prélèvement [4,6]. Dans une étude réalisée au Burkina-Faso, sur 1683 BAB, seulement, 4,2% étaient conformes aux règles de bonne prescription médicale [6]. Selon les mêmes auteurs, le coût global des BAB non conformes s´élève 14674,98 $ US soit 8 394 088,56 FCFA [6].

Au Niger, à notre connaissance, aucune étude n´a porté sur la qualité rédactionnelle des BAB. L´objectif général de cette étude était d´évaluer la qualité rédactionnelle des BAB dans les principales structures de santé et laboratoire du Niger. De manière spécifique, il s´est agi de: (i) analyser le niveau de complétude des BAB; (ii) déterminer le coût global des BAB et les coûts moyens des BAB conformes et non-conformes; (iii) identifier les facteurs associés ou non aux BAB conformes et non-conformes.

## Méthodes

**Cadre d´étude:** il s´agit d´une étude multicentrique effectuée dans les hôpitaux et laboratoires de santé de Niamey suivant: Hôpital Général de Référence (HGR); Hôpital National de Niamey (HNN); Hôpital National Amirou Boubacar Diallo (HNABD); La Maternité Centrale Issaka Gazobi (MIG) et la représentation du laboratoire CERBA au Niger. Le choix de ces centres était motivé par le fait qu´ils constituent les principales structures hospitalières de santé publique à Niamey. La représentation du laboratoire CERBA au Niger a été rajoutée pour évaluer les BAB des échantillons envoyés hors du Niger pour être analysés.

**Type et période de l´étude:** il s´agissait d´une étude transversale, réalisée sur une période de huit (8) mois, allant du 31 mai 2021 au 31 janvier 2022.

**Critères d´inclusion:** tous les bulletins d´analyses biologiques adressés aux laboratoires retenus durant la période de collecte des données ont été inclus dans cette étude.

**Critères d´exclusion:** tous les bulletins d´analyses biologiques dupliqués aux laboratoires pour séparer les analyses de biochimie, de bactériologie et d´hématologie ont été exclus de l´étude.

**Variables de l´étude:** les informations consignées sur le BAB, décrites précédemment par Yacouba *et al*., 2019 avaient constitué les variables de l´étude. Brièvement, il s´agissait des informations portant sur le patient, le prescripteur, la nature des examens demandés et celui du prélèvement (Tableau 1).

**Tableau 1:** informations recherchées sur chaque bulletin d´analyse biologique

Informations		
Sur le patient	Sur l´examen / prélèvement	Sur le prescripteur
Identité*	Libellé exact de(s) examen(s) demandé(s)	Identité
Age	Date de prescription	Qualification
Sexe	Nature de prélèvement	Signature
Renseignements cliniques**	Date de prélèvement	Cachet
Contact	Heure du prélèvement	Contact
Profession		Numéro d´ordre
Provenance		
Numéro du lit		

* Nom et prénom du patient **Symptômes, diagnostic, traitement en cours, état physiologique

### Définitions opérationnelles

***La qualité rédactionnelle:*** correspond à l´ensemble des caractéristiques d´un BAB qui lui confèrent l´aptitude à satisfaire les exigences de bonnes prescriptions médicales. En d´autres termes, c´est le respect de l´orthodoxie de la prescription médicale selon la norme ISO 15189 [4].

***Bulletin d´analyses biologiques conforme:*** un BAB était déclaré conforme lorsqu´il portait tous les renseignements suivants: les informations sur le patient (identité, âge, sexe), le service demandeur, les informations sur le prescripteur (identité, signature, qualification et cachet), les renseignements cliniques et thérapeutiques, la date de prescription et la nature de l´échantillon.

***Bulletin d´analyses biologiques non-conforme:*** correspond au BAB sur lequel il manque une ou plusieurs informations définissant un BAB conforme.

**Collecte des données:** la collecte des données a été réalisée au niveau des différents sites (HGR, HNN, HNABD, MIG, CERBA-Niger) de l´étude en utilisant une fiche de collecte effectuée avec le logiciel Epi info (Version 7.2.4.0); les informations collectées étaient résumées dans le Tableau 1.

**Analyse statistique des données:** l´analyse statistique des données a été réalisée avec le logiciel Epi info (Version 7.2.4.0) et RStudio (Version 4.1.1). Les BAB portant ou non l´identité du patient, le service demandeur, l´identité du prescripteur, la date, l´heure et la nature du prélèvement, la signature et le cachet du prescripteur, ont été décrits sous forme des effectifs et pourcentages. Les BAB ont été catégorisés en BAB conformes et non-conformes selon qu´ils satisfont ou non aux exigences de bonnes prescriptions médicales. Le coût global des BAB inclus et les coûts moyens (avec écart-type) des BAB conformes et non-conformes ont également été déterminés. Le test de de khi^2^ de Pearson ou la probabilité exacte de Fisher (lorsque les conditions de validité de test de de khi^2^ ne sont pas remplies) a été réalisé pour comparer la proportion des différentes variables en fonction de la conformité ou non des BAB. Le test de Student a été utilisé pour comparer les coûts moyens des BAB conformes et non-conformes. Une analyse logistique multivariée a été réalisée pour déterminer les facteurs associés ou non aux BAB conformes et non-conformes. Une valeur p < 0.05 a été considérée comme significative.

**Considérations éthiques:** une autorisation des directeurs des différents centres de santé retenus pour l´étude, ainsi que l´accord des chefs des services des différents laboratoires respectifs avaient été demandés et obtenus avant le début de cette étude. Aussi, l´anonymat des patients et des prescripteurs a été respecté.

## Résultats

### Fréquence de notification des informations requises sur les bulletins d´analyses biologiques

**Figure 1:**
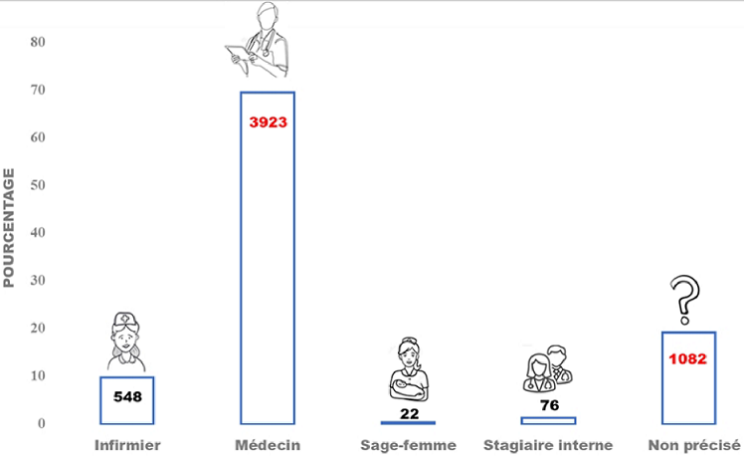
répartition des prescripteurs en fonction de la qualification

Au total, 5 651 BAB ont été inclus dans cette étude et proviennent majoritairement de l´HNN (n=2019; 35,73%) et de l´HNABD (n=2008; 35,53%). Sur les 5 651 BAB inclus, 53,99% (n=3 051) ne portaient pas le nom du service demandeur. Parmi les BAB portant le nom du service demandeur (n=2600), les services des urgences médicales et celui de la pédiatrie/CRENI étaient le plus représentés avec, respectivement, 5,20% (n=294) et 4,78% (n=270) (Tableau 2). Sur les 5 651 BAB inclus, 19,15% (n=1082) ne portaient pas la qualification du prescripteur. Parmi les BAB portant la qualification du prescripteur, les médecins représentaient 69,42% (n=3 923) des prescripteurs, suivis des infirmiers, 9,70% (n=548) (Figure 1). Les noms, prénoms des patients et la date de prescription étaient les informations les plus notifiées sur les BAB avec respectivement 99,79% (n=5 639), 99,65% (n=5 631), et 97,45% (n=5 507). A contrario, les informations les plus négligées sur les BAB étaient la date, l´heure et la nature du prélèvement représentant respectivement 0,02% (n=1), 0,21% (n=12) et 1,68% (n=95) des notifications (Tableau 3). Bulletins d´analyses biologiques conformes Globalement, sur 5 651 BAB inclus dans cette étude, seulement 534 (9,45%) étaient conformes aux règles de bonne prescription des BAB. Il existe une différence statistiquement significative de la conformité des BAB en fonction des centres de santé (p-value = 0,00001) (Tableau 4). Cependant, la proportion des BAB conformes étaient comparables entre les services médicaux et chirurgicaux. Aussi, il existe une différence statistiquement significative de la conformité des BAB en fonction de la qualification du prescripteur. Les médecins ont tendance à prescrire mieux que les autres agents de santé avec 13,56% (n=532) des prescriptions conformes contre 0,36% (n=2) pour les infirmiers (Tableau 5).

**Tableau 2:** répartition des BAB en fonction des services demandeurs

Service demandeur	Fréquence	Pourcentage %
**Anesthésie - Réanimation**	17	0,30%
**Bloc opératoire**	89	1,57%
**Cardiologie**	176	3,11%
**Chirurgie générale et viscérale**	86	1,52%
**Chirurgie pédiatrique**	42	0,74%
**Dermatologie / allergologie**	19	0,34%
**Endocrinologie / Nutrition / Diététique**	63	1,11%
**Gynécologie**	22	0,39%
**Hépato-gastro-entérologie**	39	0,69%
**Médecine Interne**	205	3,64%
**Non précisé**	**3051**	**53,99%**
**Néonatologie**	62	1,10%
**Néphrologie**	217	3,84%
**Neurochirurgie**	18	0,32%
**Neurologie**	106	1,88%
**Obstétrique**	40	0,71%
**Oncohématologie**	31	0,55%
**Ophtalmologie**	44	0,78%
**ORL / Chirurgie cervico-faciale**	50	0,88%
**Pavillon de la Francophonie**	15	0,27%
**Pavillon Raymond Madras**	37	0,65%
**Pédiatrie/ CRENI**	**270**	**4,78%**
**Pneumologie**	88	1,56%
**Psychiatrie**	3	0,05%
**Rhumatologie**	53	0,94%
**Stomatologie / Chirurgie Maxillo- faciale**	18	0,32%
**Traumatologie-Orthopédie**	132	2,33%
**Urgence chirurgicale**	21	0,37%
**Urgence médicale**	**294**	**5,20%**
**Urgence pédiatrique**	268	4,74%
**Urologie**	75	1,33%
**TOTAL**	**5651**	**100,00%**

**Tableau 3:** information requise sur les BAB et fréquences de leur notification

Critères	Existence			
	Non		Oui	
	Effectifs	Pourcentage %	Effectifs	Pourcentage %
**Nom du patient**	12	0,21	5639	99,79
**Prénom du patient**	20	0,35	5631	99,65
**Âge du patient**	639	11,31	5012	88,69
**Sexe du patient**	2200	38,93	3451	61,07
**Identité du prescripteur**	2181	38,59	3470	61,41
**Signature du prescripteur**	1735	30,70	3916	69,30
**Qualification du prescripteur**	1082	19,15	4569	80,85
**Cachet du prescripteur**	1543	27,30	4108	72,70
**Contact prescripteur**	5497	97,27	154	2,73
**Numéro d´ordre du prescripteur**	4759	84,22	892	15,78
**Date de prescription**	144	2,55	5507	97,45
**Renseignement clinique**	2635	46,63	3016	53,37
**Nature de l´echantillon**	5556	98,32	95	1,68
**Date de prélèvement**	5650	99,98	1	0,02
**Heure de prélèvement**	5639	99,79	12	0,21

**Tableau 4:** conformité des BAB en fonction des centres de santé

Centres	BAB Conformes		Total
	Non	Oui	
**CERBA**	75(75%)	25(25%)	100(100%)
**HGR**	990(96,96%)	31(3,04%)	1021(100%)
**HNABD**	1681(83,72%)	327(16,28%)	2008(100%)
**HNN**	1880(93,12%)	139(6,88%)	2019(100%)
**MIG**	491(97,61%)	12(2,39%)	503(100%)
**TOTAL**	**5117(90,55%)**	**534(9,45%)**	(**100%)**

BAB = Bulletins d´analyses biologiques conformes. CERBA: Représentation du Laboratoire CERBA au Niger. HGR: Hôpital Général de Reference. HNABD: Hôpital National Amirou Boubacar Diallo. HNN: Hôpital National de Niamey. MIG: Maternité Issaka Gazobi

**Tableau 5:** conformité des BAB en fonction de la qualification du prescripteur

Qualification	Conforme		TOTAL
	Non	Oui	
**Infirmier**	546(99,64%)	2(0,36%)	548(100%)
**Médecin**	3391(86,44%)	532(13,56%)	3923(100%)
**Sage-femme**	22(100%)	0(0%)	22(100%)
**Stagiaire interne**	76(100%)	0(0%)	76(100%)
**TOTAL**	**4035(88,31%)**	**534(11,69%)**	**4569(100%)**

### Coût des bulletins d´analyses biologiques

Le coût global des BAB était de 52 412 095 francs CFA, avec une moyenne de 9276,47 ± 11246,04 francs CFA. Le coût moyen des BAB conformes (9051,18 francs CFA) était statistiquement comparable à celui des BAB non-conformes (9299,99 francs CFA) (p-value =0,62665).

### Analyse multivariée

**Figure 2:**
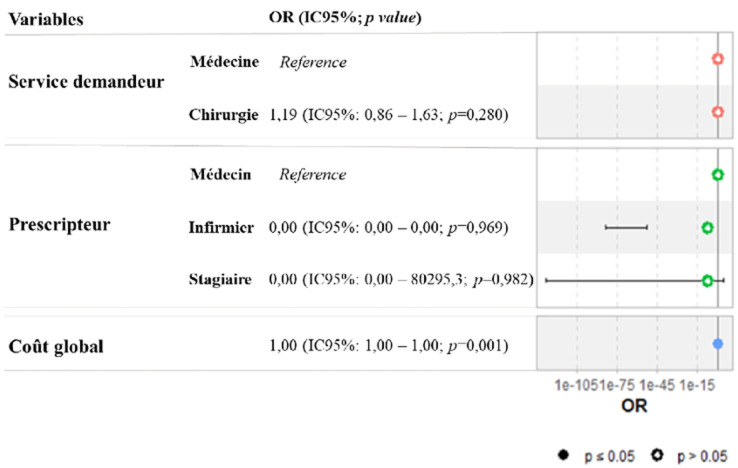
régression logistique multivariée pour déterminer les facteurs de risque des bulletins d´analyses biologiques non conformes entre les services demandeurs, la qualification et le coût global des analyses biologiques demandées

À l´analyse logistique multivariée, aucune augmentation du risque des BAB conformes n´était observée en fonction de la qualification du prescripteur, du service demandeur, et du coût des analyses (Figure 2).

## Discussion

La rédaction du BAB, étape initiale de la phase pré-analytique, amorce le début d´une analyse biologique. La prescription d´un BAB comporte un certain nombre d´informations sur le prescripteur ainsi que son service, sur le patient (de son identité jusqu´aux renseignements cliniques), et sur le prélèvement. Une insuffisance dans le renseignement d´un item conduit à une non-conformité du BAB et peut avoir un impact direct sur le rendu du résultat et donc sur la qualité de soin fourni au patient. Dans la littérature, 68,2% des erreurs de laboratoire étaient attribuées à la phase pré-analytique [7]. Le but de cette étude était d´évaluer la qualité rédactionnelle et le coût des BAB dans les principales structures de santé de Niamey. Au total, 5 651 BAB ont été inclus dans cette étude et provenaient majoritairement de l´HNN (n=2019; 35,73%) et de l´HNABD (n=2008; 35,53%). Ceci pourrait s´expliquer par le fait que ces deux centres de santé sont les plus anciens de la commune urbaine de Niamey et surtout les mieux connus par la population nigérienne. Par leur proximité par rapport aux autres centres de soins, ils reçoivent plus d´évacuation sanitaire et d´urgence.

Les informations les plus renseignées sur les BAB étaient l´identité du patient (99,79%), la date de la prescription (97,45%), et l´âge (88,69%) des patients. Le même constat était observé par Oyedeji *et al*., au Nigéria en 2015 [8], Yacouba *et al*., au Burkina Faso en 2019 [6], Olayemi *et al*., au Ghana en 2012 [9] et Singh *et al*., au Népal en 2015 [10], qui avaient rapporté respectivement 99,0%, 99,8%, 100% et 100% des BAB portant l´identité du patient. Une étude réalisée au Burkina Faso avait rapporté que 96,7% des BAB renseignaient l´âge du patient [6]. Compte tenu des valeurs de référence biologique, l´âge et le sexe trouvent leur importance pour certains paramètres, favorisant ainsi une meilleure interprétation des résultats.

Parmi les informations le moins notifiées, on retrouve la date du prélèvement (0,02%), suivie de l´heure du prélèvement (0,21%), de la nature du prélèvement (1,68%), et du contact du prescripteur (2,73%). Dans la logique de nos résultats, Yacouba *et al*., 2019 [6], avait rapporté une notification du contact du prescripteur dans seulement 1% des BAB tandis que Olayemi *et al*., 2012 [9], et Oladeinde *et al*., 2012 [11] n´avaient rapporté aucun BAB avec le contact du prescripteur. Cette situation rend très difficile la communication des urgences diagnostiques et des résultats critiques. La nature de l´échantillon biologique est essentielle dans la détermination de la méthode et du matériel de prélèvement. Aussi, faut-il ajouter que l´adaptation du récipient destiné à contenir l´échantillon en vue de déterminer le mode de transport et de conservation avant l´analyse en dépend. En cas de prélèvement des liquides biologiques sanguinolents (urine, liquide pleural, liquide d´ascite, liquide cérébro-spinal, etc…), mentionner la nature de l´échantillon est très important. Et pour cause, ces liquides pouvant être confondus avec le sang, si précision n´y est pas; cela peut entrainer l´utilisation des valeurs de référence non adaptées pour l´interprétation des résultats [2].

Par ailleurs, l´absence des services demandeurs sur les BAB, représentait 53,99% dans notre étude. Ce résultat est supérieur à ceux de Oladeinde *et al*., 2012 [11] et Olayemi *et al*., 2012 [9] qui ont obtenu respectivement 20,1% et 47.8%. Par contre, ce résultat était inférieur à celui obtenu (61,39%) par Gyawali *et al*., au Népal en 2016 [12]. L´absence du service demandeur sur la feuille de prescription ne permet pas au biologiste de rechercher des informations complémentaires sur les conditions de réalisation du prélèvement ou d´une demande d´un autre prélèvement, si le prélèvement initial n´est pas adéquat. De même, il est très difficile pour le laboratoire de communiquer avec le service en cas de panne ou de retard dans le délai de rendu des résultats. En plus, la précision du service demandeur sur les BAB permettra au laboratoire d´identifier rapidement les services produisant plus de non-conformité et prendre les mesures correctives.

Dans notre étude, la qualification du prescripteur était notifiée dans 80,85% des BAB. Les médecins représentaient 69,42% des prescripteurs, suivis des infirmiers, 9,70%. Nos résultats sont différents de ceux obtenus par Yacouba *et al*., 2019 [6] qui ont rapporté que les stagiaires en médecine représentaient 71,4% des prescripteurs et les médecins 21,3%. Il est très important que le prescripteur finalise sa prescription médicale en apposant sa signature sur le BAB. Ceci permet de confirmer son professionnalisme, mais aussi l´authenticité du BAB qu´il a émis. Dans notre étude 69,30% des prescripteurs apposaient leur signature sur les BAB. Olayemi *et al*., 2012 [9] et de Oladeinde *et al*., 2012 [11] avaient rapporté que les BAB étaient dûment signés par le prescripteur respectivement dans 75,7% et 100% des cas.

L´absence des renseignements cliniques représentait 46,63%(n=2635) des BAB dans cette étude. Ce résultat est inférieur à ceux de Yacouba *et al*., 2019 [6] qui ont rapporté 59,4%. Nos résultats étaient cependant supérieurs à ceux de Sharif *et al*., au Pakistan en 2007 [13], Olayemi *et al*., 2012 [9] et Oladeinde *et al*., 2012 [11], qui avaient rapporté respectivement 34%, 22,7% et 6,4%. Burton *et al*., 2001 avaient démontré que la notification des renseignements cliniques adéquats prévient les investigations inappropriées [14].

La conformité des différents BAB prescrits dans nos différents centres de santé a été évaluée selon les recommandations sur les règles de bonnes pratiques rédactionnelles des BAB. Les résultats de cette étude ont montré que sur un effectif de 5651 BAB, seulement 534(9,45%) étaient appréciés conformes. Ce résultat alarmant traduit une grande faiblesse de la qualité rédactionnelle des BAB au Niger. Nos résultats semblent légèrement au-dessus de ceux rapporté aux Burkina Faso où 4,2% des BAB étaient conformes aux bonnes pratiques rédactionnelles des BAB [6]. La non maitrise de la qualité rédactionnelle des BAB entraine une augmentation de la marge d´erreurs au sein du laboratoire, un retard dans le délai de la prise en charge des spécimens biologiques et dans l´établissement des diagnostics des patients. Elle ne permet pas de garantir la maîtrise exacte et opportune des résultats. Par contrecoup, la non maitrise de la qualité rédactionnelle des BAB influe négativement sur la qualité des soins et sur la sécurité à fournir aux patients.

Les examens complémentaires de biologie médicale génèrent des coûts et dépenses importants respectivement pour les laboratoires et les patients. Dans notre étude, le coût des examens biologiques prescrits sur les BAB non-conformes représentait 47 578 760 francs CFA du coût global des examens. Le coût moyen d´un BAB conforme (9051,18 francs CFA) était statistiquement comparable à celui d´un BAB non-conformes (9299,99 francs CFA). Au Burkina Faso, le coût global des BAB non conformes s´élevait à 8 394 088,56 francs CFA [6]. Limites de l´étude: Les structures évaluées étant les principaux centres de référence des toutes les spécialités médicales et chirurgicales, les résultats peuvent être surestimés à l´échelle du pays. Aussi, la non-prise en compte de l´impact de la non-conformité des BAB sur la prise en charge des patients constitue également une autre limite de cette étude.

## Conclusion

Les résultats de cette étude avaient démontré que la qualité rédactionnelle des BAB est très faible dans les structures de santé évaluées. Si les médecins ont tendance à prescrire mieux que les autres agents de santé à l´analyse bivariée, l´analyse multivariée avait montré que le risque des BAB non conformes n´était pas associé à la qualification du prescripteur, au service demandeur, et au coût des analyses. Une attitude rigoureuse au niveau des laboratoires est nécessaire pour la maitrise, la gestion et le traitement des non-conformités. Ces résultats soulignent également la nécessité d´un dialogue clinico-biologique pour une meilleure utilisation des résultats des laboratoires, en réduisant les délais de rendu des résultats et en évitant les reprises des examens non-conformes à l´origine des dépenses supplémentaires pour les patients. Un laboratoire qui s´engage dans cette rigueur ne peut qu´être performant en jouant son rôle régalien d´outil de décision incontournable pour la médecine moderne.

### Etat des connaissances sur le sujet

- Le bulletin d´analyses biologiques est un moyen de communication entre le clinicien qui demande un examen et le biologiste qui le réalise et l´interprète;
- L´absence de rigueur dans l´élaboration des bulletins d´analyses biologiques peut conduire à une non-conformité dont la finalité est le rejet de l´examen; elle est également à l´origine du retard de prise en charge du patient et une perte de crédibilité de la prestation du laboratoire.

### Contribution de notre étude à la connaissance

- Cette étude a évalué la qualité rédactionnelle des bulletins d´analyses biologiques dans plusieurs hôpitaux et laboratoire de référence du Niger;
- Ce travail a démontré que la qualité rédactionnelle des BAB est très faible dans les structures de santé évaluées;
- Aussi, cette étude a démontré que le risque des BAB conformes ou non-conformes n´était pas associé à la qualification du prescripteur, au service demandeur, et au coût des analyses.

## Remerciements

Toute notre gratitude à l´endroit des directeurs des différents centres de santé retenus pour l´étude, ainsi qu´aux personnels des différents laboratoires respectifs pour leurs différentes collaborations.
